# The Merkel Cell Polyomavirus T-Antigens and IL-33/ST2-IL1RAcP Axis: Possible Role in Merkel Cell Carcinoma

**DOI:** 10.3390/ijms23073702

**Published:** 2022-03-28

**Authors:** Kashif Rasheed, Ugo Moens, Benedetta Policastro, John Inge Johnsen, Virve Koljonen, Harri Sihto, Weng-Onn Lui, Baldur Sveinbjørnsson

**Affiliations:** 1 Molecular Inflammation Research Group, Department of Medical Biology, Faculty of Health Sciences, University of Tromsø, 9037 Tromsø, Norway; ugo.moens@uit.no (U.M.); be.policastro@studenti.unina.it (B.P.); baldur.sveinbjornsson@uit.no (B.S.); 2 Childhood Cancer Research Unit, Department of Women’s and Children’s Health, Karolinska Institute, 17177 Stockholm, Sweden; john.inge.johnsen@ki.se; 3 Department of Plastic Surgery, University of Helsinki and Helsinki University Hospital, 00280 Helsinki, Finland; virve.koljonen@hus.fi; 4 Department of Pathology, University of Helsinki, 00100 Helsinki, Finland; harri.sihto@helsinki.fi; 5 Department of Oncology-Pathology, Karolinska Institute, BioClinicum, Karolinska University Hospital, 17164 Solna, Sweden; weng-onn.lui@ki.se

**Keywords:** cytokines, IL-33, Merkel cell carcinoma, inflammation, ST2/IL1RL1, IL1RAcP, tumor microenvironment

## Abstract

Merkel cell polyomavirus (MCPyV) is a causal factor in Merkel cell carcinoma (MCC). The oncogenic potential is mediated through its viral oncoproteins large T-antigen (LT) and small T-antigen (sT). Cytokines produced by tumor cells play an important role in cancer pathogenesis, and viruses affect their expression. Therefore, we compared human cytokine and receptor transcript levels in virus positive (V+) and virus negative (V−) MCC cell lines. Increased expression of IL-33, a potent modulator of tumor microenvironment, was observed in V+ MCC cell lines when compared to V− MCC-13 cells. Transient transfection studies with luciferase reporter plasmids demonstrated that LT and sT stimulated IL-33, ST2/IL1RL1 and IL1RAcP promoter activity. The induction of IL-33 expression was confirmed by transfecting MCC-13 cells with MCPyV LT. Furthermore, recombinant human cytokine domain IL-33 induced activation of MAP kinase and NF-κB pathways, which could be blocked by a ST2 receptor antibody. Immunohistochemical analysis demonstrated a significantly stronger IL-33, ST2, and IL1RAcP expression in MCC tissues compared to normal skin. Of interest, significantly higher IL-33 and IL1RAcP protein levels were observed in MCC patient plasma compared to plasma from healthy controls. Previous studies have demonstrated the implication of the IL-33/STL2 pathway in cancer. Because our results revealed a T-antigens-dependent induction of the IL-33/ST2 axis, IL-33/ST2 may play a role in the tumorigenesis of MCPyV-positive MCC. Therefore, neutralizing the IL-33/ST2 axis may present a novel therapeutic approach for MCC patients.

## 1. Introduction

Merkel cell carcinoma (MCC) is a rare but highly aggressive neuroendocrine skin cancer associated with Merkel cell Polyomavirus (MCPyV; 80% of the cases) or UV exposure [1,2,3]. MCPyV is a non-enveloped, double-stranded DNA virus with a circular genome of ~5 kb [4]. The viral genome is divided into three major regions: the non-coding control region (NCCR), the early region, and the late region. The NCCR contains the origin of replication, as well as the early and late promoters that control expression of the early region and the late region, respectively. The early region encodes large T-antigen (LT), small T-antigen (sT), 57 kT, and a protein called alternative LT open reading frame (ALTO) [5,6,7,8]. While the oncogenic potential of LT and sT are well documented, the roles of 57 kT and ALTO in the viral life cycle and MCC tumorigenesis remain elusive [9]. The late region encodes for the viral capsid proteins VP1 and VP2 [10]. The MCPyV genome is maintained as episomal circular dsDNA during the normal life cycle but is integrated in virus positive (V+) MCCs [11]. Characteristic for V+ MCC is the expression of a C-terminal truncated LT (tLT), resulting from mutations or a reading frame shift, which introduce premature stop codons [7,12,13,14].

Inflammatory pathways have been associated with tumor progression and therapy resistance [15,16]. Cancers may arise from sites of chronic irritation and inflammation, while oncogenic mutations may activate inflammatory signal transduction cascades [17,18]. Cytokines are central mediators in an inflammatory tumor microenvironment. Interleukin-33 (IL-33) is considered as an “alarmin” (endogenous danger signal) released following cellular damage [19] and belongs to the IL-1 family of cytokines that are expressed in multiple organs and cell types in both humans and mice. It is the ligand for the “suppression of tumorigenicity 2 L receptor” (ST2) and “IL-1 receptor accessory protein” (IL1RAcP) [20].

IL-33 induces cytokine and chemokine expression in various cell types and tissues [21]. Aberrant expression of IL-33 and its receptors has been described for multiple types of cancer [20,22], and has also been linked to increased malignancy and worse prognoses [23,24]. Of interest, a recent study has reported a protection against osteosarcoma tumorigenesis as a result of functional polymorphism in the IL-33 promoter region [25]. Several reports on IL-33 have described a wide variety of effects attributed to tumor growth, such as increased cell adhesion, invasion, survival, and proliferation [19,20,22,26]. Furthermore, IL-33 affects the tumor microenvironment (TME) through immune cells, such as myeloid-derived suppressor cells (MDSCs), dendritic cells (DCs), and regulatory T cells (Tregs) [19]. We have previously reported an increased expression of the C-C motif ligand 17/thymus and activation-regulated chemokine in V+ MCC compared to V− MCC [27]. In the current study, we examined the effect of MCPyV T-antigens (T-ags) on IL-33 expression and its functional role in MCC.

## 2. Results

### 2.1. Differential Expression of Inflammatory Cytokines and Receptors in MCC Cell Lines

**Figure 1:**
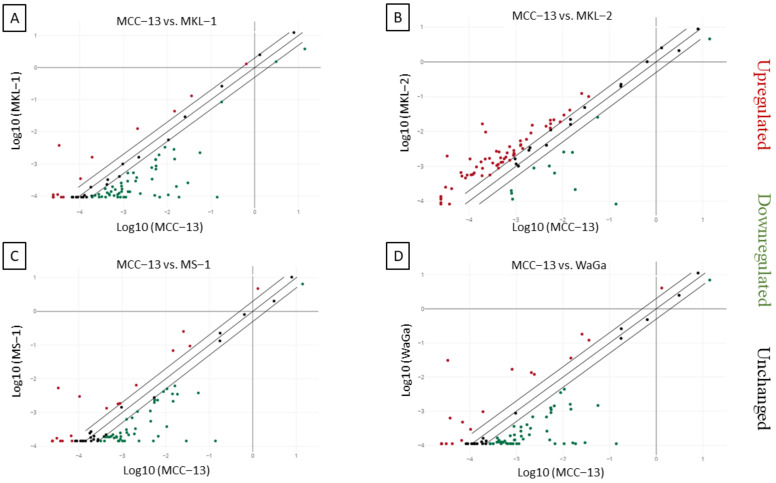
Relative expression comparison of 84 inflammatory cytokines and receptors genes between MCPyV-positive and negative Merkel cell carcinoma cell lines. The figure depicts a log transformation plot of the relative expression level differences for each gene (2^−ΔCt^) between (**A**) MCC-13 cells vs. MKL-1 cells, (**B**) MCC-13 cells vs. MKL-2 cells, (**C**) MCC-13 cells vs. MS-1 cells, and (**D**) MCC-13 cells vs. WaGa cells. The lines indicate a two-fold change in the gene expression threshold.

The RT^2^ profiler PCR array was used to access the expression pattern of different inflammatory cytokines and receptors in V+ MKL-1, MKL-2, MS-1, and WaGa MCC cell lines and in V− MCC-13 cells. Gene expression of 84 different genes was determined, with a gene considered as differentially expressed when the expression level exceeded two-fold in all the cell lines examined. Most of the genes examined were detectable (MCC-13 82/84, MKL-1 83/84, MKL-2 83/84, MS-1 80/84, WaGa 81/84) with Cq values ˂ 35. Compared to MCC-13 cells, the number of differentially expressed genes was 65 for MKL-1 (13 upregulated, 52 downregulated), 71 for MKL-2 (60 upregulated, 11 downregulated), 63 for MS-1 (18 upregulated, 45 downregulated), and 70 for WaGa (19 upregulated, 51 downregulated) (Figure 1A–D). Overall, the data demonstrated that 22 different inflammatory cytokines and receptors were differentially expressed (12 upregulated, 10 downregulated) between all V+ cell lines and the V− MCC-13 cell line.

### 2.2. Increased IL-33 Expression in MCPyV Positive-MCC Cell Lines Compared to Virus Negative-MCC Cell Lines

**Figure 2:**
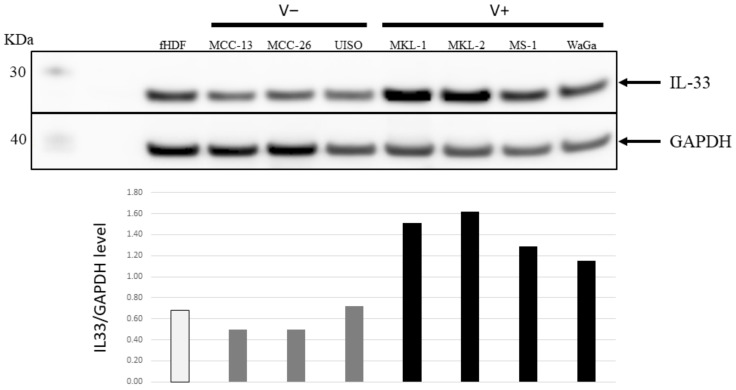
IL-33 expression levels in V− and V+ MCC cell lines. Top panel: Western blot analysis of IL-33 expression in V− MCC (MCC-13, MCC-26, and UISO) and V+ MCC (MKL-1, MKL-2, MS-1, and WaGa) cell lines. Immortalized human dermal fibroblasts (fHDF) were used as control cells and GAPDH was used as a loading control. Bottom panel: ratio of the values obtained by densitometric scanning of the signals obtained with IL-33 and GAPDH antibodies.

One of the genes with higher transcript levels in virus-positive cells, compared with virus-negative cells, was the IL-33 gene. We decided to focus on this cytokine because its role in viral infections (including tumor viruses), immunoresponses, and cancer is well established [20,28,29,30,31,32,33]. To confirm this at the protein level, western blots were performed on lysates from V+ and V− cell lines using an IL-33-specific antibody. The result revealed a two- to three-fold higher IL-33 levels in the V+ cell lines (MKL-1, MKL-2, MS-1, and WaGa) in comparison to the V− cell lines (MCC-13, MCC-26, and UISO), and the hTERT-immortalized human dermal fibroblast cell line fHDF/TERT166 (Figure 2).

### 2.3. MCPyV T-Antigens Stimulates IL-33 Promoter Activity and Increases IL-33 Expression

**Figure 3:**
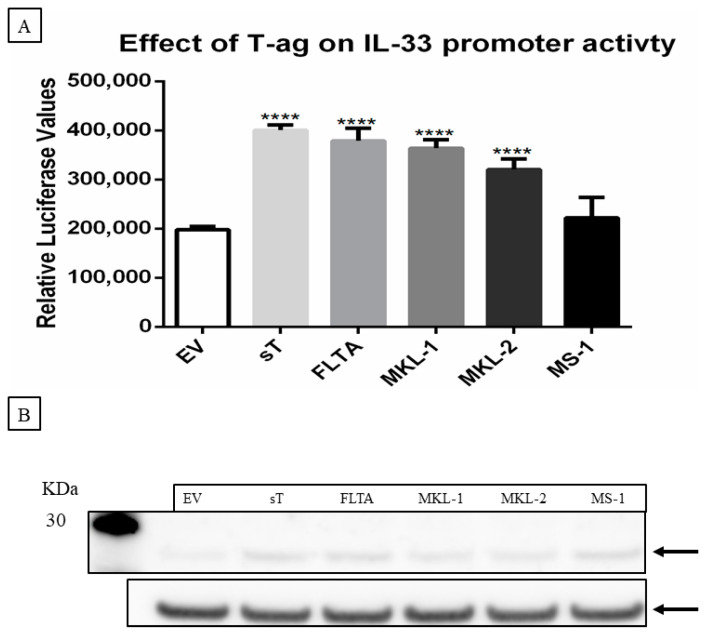

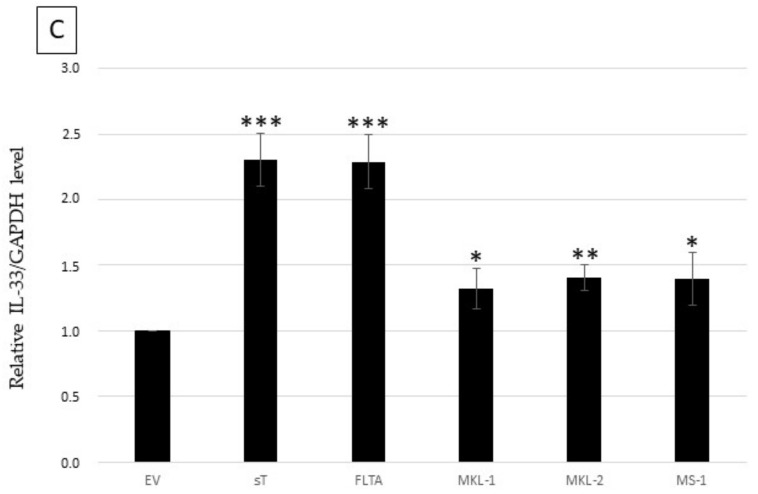
MCPyV T-antigens stimulate *IL-33* promoter activity and induce IL-33 expression levels. (**A**) MCC-13 cells were co-transfected with a luciferase reporter vector driven by the *IL-33* promoter fragment spanning the nucleotides −1050/+50, together with an expression plasmid for full-length LT (FLTA), tLT (LT_MKL-1_, LT_MKL-2_, LT_MS-1_), sT, or pcDNA3 (empty vector; EV). Luciferase activity was assessed 24 h after transfection. Each bar represents the average of three independent parallels ± SD. Luciferase values were normalized for total protein in each sample. ** *p* ≤ 0.01, *** *p* ≤ 0.001 and **** *p* ≤ 0.0001. (**B**) MCC-13 cells were transfected with expression plasmids for LT or tLT, sT, or empty vector, and protein expression was measured at 24 h after transfection. IL-33 expression was normalized with GAPDH. The image is representative for three independent experiments. (**C**) Ratio of the values obtained by densitometric scanning of the signals obtained with IL-33 and GAPDH antibodies. The ratio for empty vector:GAPDH was arbitrarily set as 1.0 ± standard deviation (* *p* < 0.05; ** *p* < 0.01; *** *p* < 0.001; ****p<0,0001).

Hallmarks of V+ MCC tumors and cell lines are the integration of the MCPyV genome and the presence of a nonsense mutation in the LT gene encoding a C-terminal truncated form of LT (tLT) [7,12,13,14]. We investigated whether full-length LT (LT), tLT variants, and sT could induce the expression of IL-33. Therefore, MCC-13 cells were transiently co-transfected with a luciferase reporter plasmid driven by the *IL-33* promoter and an expression plasmid for either MCPyV LT, three tLT variants (LT_MKL-1_, LT_MKL-2_, LT_MS-1_), or sT. The pcDNA3.1 empty vector (EV) was used as a control plasmid. The MCPyV sT, LT, and tLT variants significantly upregulated *IL-33* promoter activity (Figure 3A), and increased IL-33 protein levels in MCC-13 cells at 24 h post-transfection (Figure 3B). These results show that full-length and tLT, as well as sT, can increase the transcriptional activity of the *IL-33* promoter and enhance IL-33 expression levels.

**Figure 4:**
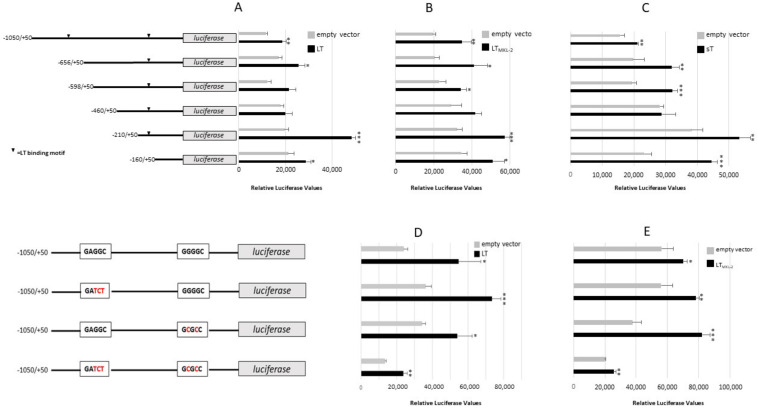
Effect of MCPyV LT and sT on the activity of *IL-33* promoter mutants. (**A**–**C**) MCC-13 cells were co-transfected with a luciferase reporter plasmid containing the human *IL-33* promoter encompassing sequences −1050 to +50 or truncated version, together with an empty expression vector pcDNA3.1 (EV) or an expression of plasmid encoding MCPyV full-length LT (**A**), LT_MKL-2_ (**B**) or sT (**C**). (**D**,**E**) MCC-13 cells were co-transfected with a luciferase reporter plasmid containing *IL-33* promoter sequences −1050/+50, or with a mutation of either one or both potential LT binding sites, together with empty vector, LT (**D**) or LT_MKL-2_ (**E**). Mutated sequences are indicated in red. Luciferase values were normalized to the total protein concentration in the sample. Each bar represents the average ± SD of three independent parallels. Each experiment was performed 2 to 4 times, and similar results were obtained. * *p* < 0.05, ** *p* < 0.01; *** *p* < 0.001.

To identify which sequences in the IL-33 promoter mediated LT- and sT-stimulated promoter activity, we generated luciferase reporter plasmids with truncated *IL-33* promoter sequences. The LT of human polyomaviruses has been shown to bind directly to DNA through GRGGC motifs [34]. The *IL-33* promoter contains two predicted MCPyV LT binding sites at position −821/−817 and −199/−195, thereby suggesting that LT may stimulate *IL-33* promoter activity by binding to these sites. The tLT versions lack their DNA binding domain and are unable to bind directly to DNA [7]. However, all three tLT variants enhanced *IL-33* promoter activity. To further ensure that LT-triggered stimulation of the *IL-33* promoter was independent of direct binding, we deleted one or both LT motifs in the *IL-33* promoter. MCC-13 cells were transiently co-transfected with pGL3_IL-33 (−1050/+50), pGL3_IL-33 (−656/+50), pGL3_IL-33 (−598/+50), pGL3_IL-33 (−460/+50), pGL3_IL-33 (−210/+50), or pGL3_IL-33 (−160/+50) luciferase plasmids, and with either of the LT, LT_MKL-2_, or sT expression plasmids. The truncated promoter (−160/+50) had a significantly higher basal activity than the (−1050/+50) promoter. Nevertheless, the activity of truncated promoter fragments was still increased by LT, LT_MKL-2_, and sT (Figure 4A–C). The mutation of either one or both LT binding motifs did not abrogate the stimulation of the *IL-33* promoter activity by LT or tLT (Figure 4D,E). There was no significant difference between LT- and LT_MKL-2_-induced transactivation of the different *IL-33* promoter fragments (*p* varied between 0.1232 to 0.9020), except for the −656/+50 construct, which was stronger activated by LT_MKL-2_ than by LT (*p* = 0.008). Although both LT and LT_MKL-2_ potentiated the transcriptional activity of the IL-33 promoter with single or double mutated LT binding motifs, induction of the promoter with mutation of the most upstream LT binding motif or of both motifs was significantly stronger by LT (*p* = 0.0260 and 0.0144, respectively) than by LT_MKL-2_, whereas this truncated LT variant stimulated the IL-33 promoter with mutated proximal LT binding motif better then full-length LT (*p* = 0.0212). These results indicate that the direct binding of LT to the *IL-33* promoter is not required for transactivation, and suggests that LT, LT_MKL-2_, and sT-induced activation is mediated by the most proximal promoter sequences.

### 2.4. MCPyV T-Ags Stimulate ST2/IL1RL1 and IL1RAcP Promoter Activity

**Figure 5:**
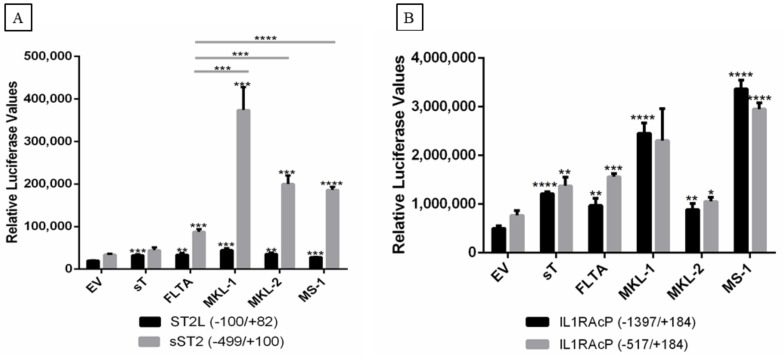
Effect of MCPyV T-ag on *ST2/IL1RL1* and *IL1RAcP* promoter activity in MCC-13 cells. Cells were co-transfected with a luciferase reporter vector driven by *ST2/IL1RL1* or *IL1RAcP* promoter sequences and full-length LT (LT), tLT (LT_MKL-1_, LT_MKL-2_, and LT_MS-1_), sT, or empty vector (EV). The promoter fragments spanning nucleotides for *ST2/IL1RL1* were −100/+82 and −499/+100 (**A**) and for *IL1RAcP* they were −1397/+184 and −517/+184 (**B**). Luciferase activity was assessed following overnight cultivation of transfected cells. Each bar represents the average of three independent parallels +SD. Luciferase values were normalized to the total protein in each sample. * *p* ≤ 0.05, ** *p* ≤ 0.01, *** *p* ≤ 0.001, and **** *p* ≤ 0.0001.

Human and mouse *ST2/IL1RL1* genes have two alternative promoter regions, the distal and proximal promoters, followed by the noncoding first exons, E1a and E1b [35]. Transcription from the distal promoter generates mRNA for the membrane-bound ST2L/IL1RL1L receptor protein, whereas the proximal promoter produces transcripts for the soluble form of the sST2/sIL1RL1 receptor [36]. To study the effect of the MCPyV T-ags on *ST2/IL1RL1* promoter regulation, we cloned the distal (pGL3_ST2L−100/+82) and the proximal promoter (pGL3_sST2−499/+100) into the luciferase reporter plasmid pGL3-basic. MCC-13 cells were co-transfected with MCPyV LT, tLT, or sT and luciferase reporter plasmid, with either the distal or proximal promoter. The results demonstrated a moderate, but significant, upregulation of the distal promoter activity by the T-ags, while a more pronounced stimulation of the proximal promoter activity was observed for all LT. No significant effect was detected for sT. Interestingly, tLT variants more potently stimulated the distal *ST2* promoter compared to full-length LT (Figure 5A).

We also evaluated the effect of MCPyV T-ags on the *IL1RAcP* promoter activity using pGL3_IL1RAcP (−1397/+184) and pGL3_IL1RAcP (−517/+184) luciferase reporter plasmids. With the exception of LT_MKL-2_, all MCPyV LT variants and sT upregulated the *IL1RAcP* promoter activity of both constructs; LT_MKL-1_ and LT_MS-1_ provoked higher *IL1RAcP* promoter activity compared to LT, LT_MKL-1_, and sT (Figure 5B).

### 2.5. Effect of IL-33, ST2, and IL1RAcP on the Activity of the MCPyV Early and Late Promoter

**Figure 6:**
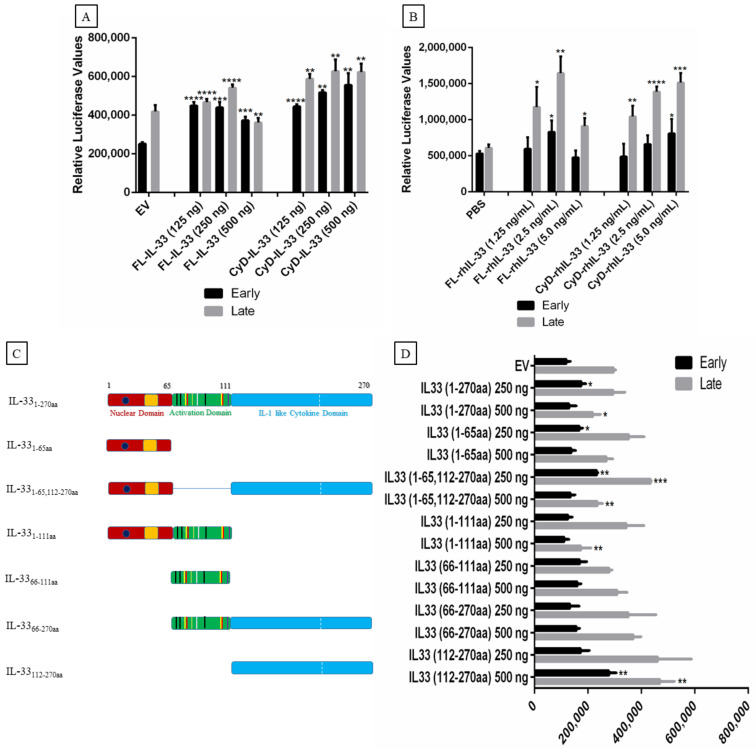

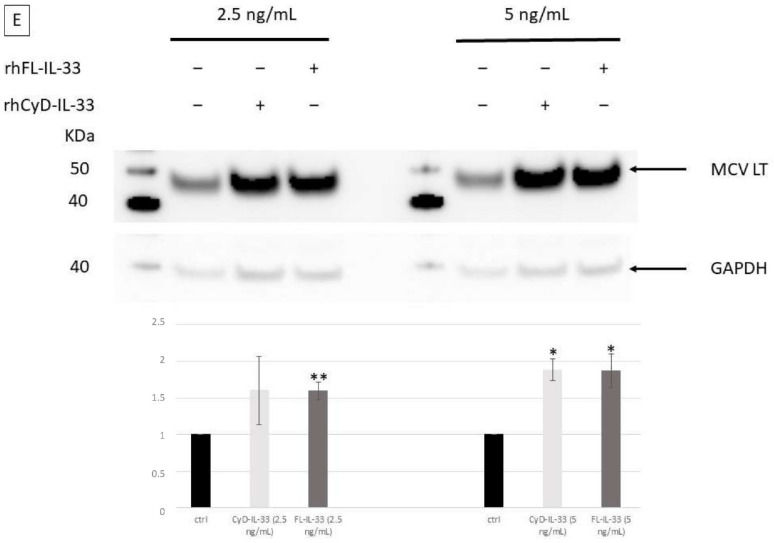
The effect of IL-33 on MCPyV promoter activity in MCC-13 cells. (**A**) Cells were transiently co-transfected with either IL-33 expression plasmid or empty expression vector pcDNA3.1, and luciferase reporter plasmid containing either the MCPyV early or the late promoter. Luciferase activity was measured 24 h after transfection. (**B**) MCC-13 cells were seeded and serum starved for 24 h. Cells were then transfected with luciferase reporter plasmid containing either the MCPyV early or the late promoter, and were exposed to FL-rhIL-33 (1.25–5.0 ng/mL), CyD-rhIL-33 (1.25–5.0 ng/mL), or vehicle (PBS) for 4 h before they were harvested and luciferase activity was measured. (**C**) Schematic presentation of the plasmids expressing the full-length or truncated forms of the IL-33 protein. Human *IL-33* mRNA (2.7 kb) encodes a protein of 270 residues with three functional domains: a nuclear, a central activation domain, and an IL-1-like cytokine domain. Sequences of the different IL-33 domains were cloned into pCMV_His(N)_FLAG(C) plasmid. (**D**) MCC-13 cells were transiently co-transfected with either MCPyV early and late promoter and 250 ng or 500 ng of plasmids expressing different domains of IL-33, IL-33_1–270aa_, IL-33_1–65aa_, IL-33_1–65_,_112–270aa_, IL-33_1–111aa_, IL-33_66–111aa_, IL-33_66–270aa_, and IL-33_112–270aa_. Abbreviations: FL-IL33 (full length IL-33), CyD-IL-33 (IL-33 with only cytokine domain). Each bar represents the average of three independent parallels + SD. * *p* ≤ 0.05, ** *p* ≤ 0.01, *** *p* ≤ 0.001, and **** *p* ≤ 0.0001. (**E**) MCPyV−positive WaGa cells were treated with recombinant full-length IL-33 (rhFL-IL-33) or with the cytokine domains, and expression levels of LT were compared with untreated cells. Levels of GAPDH were used as loading control. Cells were exposed for 16 h with the indicated concentrations of rhFL-IL-33 or rhCyD-Il-33. The experiment was performed two times and a representative result is shown. The ratio for empty vector:GAPDH from the two experiments was arbitrarily set as 1.0 ± standard deviation (* *p* < 0.05; ** *p* < 0.01).

Previous studies have demonstrated that different cytokines can modulate human polyomavirus promoter activity [27,37,38,39,40]. Therefore, we examined the effect of IL-33, ST2/IL1RL1, and IL1RAcP (both the membrane-bound and soluble forms of the receptors) on MCPyV early and late promoter activity. Co-transfection with increased concentrations of plasmid encoding full-length IL-33_1–270aa_ (FL-IL-33) increased both early and late promoter activity (Figure 6A). Stimulation of transfected cells with the recombinant cytokine domain of hIL-33 (CyD-rhIL-33) increased both early and late promoter activity in a dose-dependent manner (Figure 6A). Recombinant human FL-IL-33 (FL-rhIL-33) and CyD-IL-33 (CyD-rhIL-33) proteins also stimulated the MCPyV late promoter activity, whereas no significant effect was observed on the early promoter, with the exception of 2.5 ng/mL FL-rhIL-33 and 5.0 ng/mL CyD-rhIL-33 (Figure 6B). IL-33 has several functional domains, including a nuclear domain, an activation domain, and an IL-1-like cytokine domain (Figure 6C). To identify which domain(s) of IL-33 could activate the MCPyV promoter, we generated expression plasmids for each domain or combination of the domains. The MCPyV early promoter was significantly induced when cells were transfected with 250 ng expression plasmid encoding IL-33 fragments spanning residues (1–65), (1–270), or (1–65,112–270), whereas transfection with 500 ng expression vector for IL-33 (112–270) resulted in increased early promoter activity (Figure 6D). The late promoter was only activated by IL-33 fragments encompassing residues (1–270), (1–65,112–270), and (112–270). The highest tested concentration was required for all these expression plasmids, except for the expression plasmid IL-33 (1–65,112–270), where both concentrations significantly stimulated the late promoter activity (Figure 6D).

**Figure 7:**
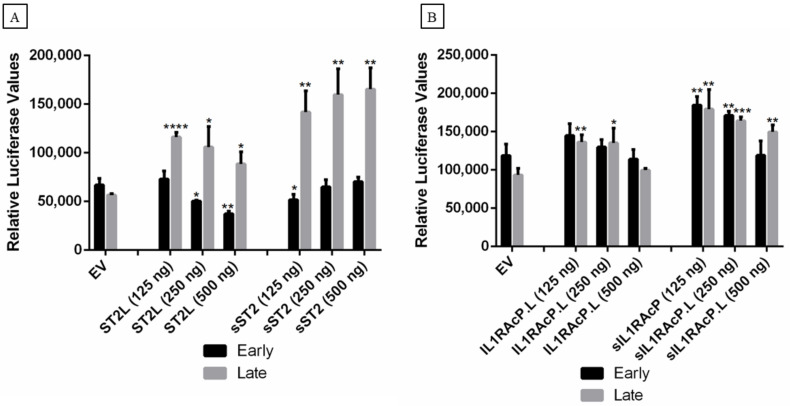
The effect of ST2/IL1RL1 and IL1RAcP on MCPyV promoter activity in MCC-13 cells. MCC-13 cells were transiently co-transfected with a luciferase reporter plasmid containing the early or late promoter of MCPyV and (**A**) ST2/IL1RL1 and (**B**) IL1RAcP expression plasmids, respectively. pcDNA3.1 was used as an empty expression vector (EV). Luciferase activity was measured at 24 h after transfection. Abbreviations: ST2L (membrane-bound ST2/IL1RL1), sST2 (soluble form of ST2/IL1RL1), IL1RAcP.L (membrane-bound IL1RAcP), sIL1RAcP (soluble form of IL1RAcP). Each bar represents the average of three independent parallels ± SD. * *p* ≤ 0.05, ** *p* ≤ 0.01, *** *p* ≤ 0.001, and **** *p* ≤ 0.0001.

We further evaluated the effect of ST2, both the membrane-bound (ST2L) and the soluble form (sST2) of the protein, on MCPyV early and late promoter activity. We show that ST2L decreased the MCPyV early but increased the late promoter activity, whereas sST2 increased the activity of the late promoter in a dose-dependent manner (Figure 7A). Moreover, we observed that sST2 had a more robust effect on late promoter compared to early promoter activity. We also investigated the effect of both the membrane-bound form and the soluble form of IL1RAcP on the MCPyV early and late promoter activity. Interestingly, we found that low concentrations of both membrane-bound and soluble forms of IL1RAcP resulted in a significant stimulation of late promoter activation, while an inhibition was observed with an increase in expression plasmid concentration. The transcriptional activity of the early promoter was only enhanced by the soluble from of IL1RAcP (Figure 7B).

### 2.6. Effect of IL-33, ST2/IL1RL1, and IL1RAcP on IL-33/ST2-IL1RAcP Complex Promoters

**Figure 8:**
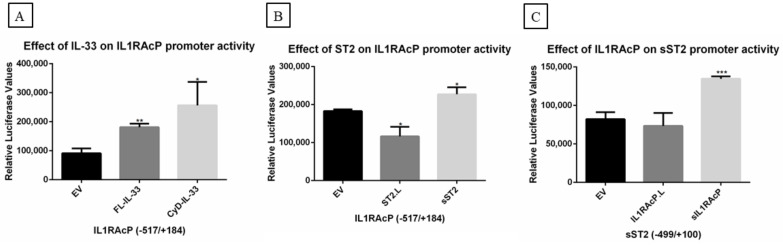
The effect of IL-33, ST2/IL1RL1, and IL1RAcP on *IL-33/ST2-IL1RAcP* complex promoters. MCC13 cells were transiently co-transfected with IL-33, ST2/IL1RL1, IL1RAcP, or the empty vector (EV) as control expression plasmids. The promoter fragments spanning nucleotides for (**A**,**B**) IL1RAcP and (**C**) sST2/sIL1RL1 were −517/+184, and −499/+100, respectively. Each bar represents the average of three independent parallels ± SD. The experiment was repeated 3 times. Luciferase values were normalized with a total protein in each sample. * *p* ≤ 0.05, ** *p* ≤ 0.01, and *** *p* ≤ 0.001.

Next, we investigated the effect of IL-33, ST2/IL1RL1, or IL1RAcP on *IL-33*/*ST2-IL1RAcP* complex promoter activity. We observed that both FL-IL-33 and CyD-IL-33 activated *IL1RAcP* promoter activity (Figure 8A). We also observed an increase or inhibition of *IL1RAcP* promoter activity by sST2/IL1RL1 and ST2.L, respectively (Figure 8B). In addition, sIL1RAcP upregulated sST2/IL1RL1 promoter activity (Figure 8C) without displaying an effect on the *ST2.L* promoter (data not shown). We did not observe an effect on IL-33 promoter activity by either ST2/IL1RL1 or IL1RAcP, and no significant effect of FL-IL-33 or CyD-IL-33 on the promoter of the membrane-bound or soluble forms of the ST2 receptor was seen (data not shown).

### 2.7. IL-33 Activates Mitogen-Activated Protein Kinase (MAP Kinase) and NF-κB Signaling Pathways in MCC Cells

**Figure 9:**
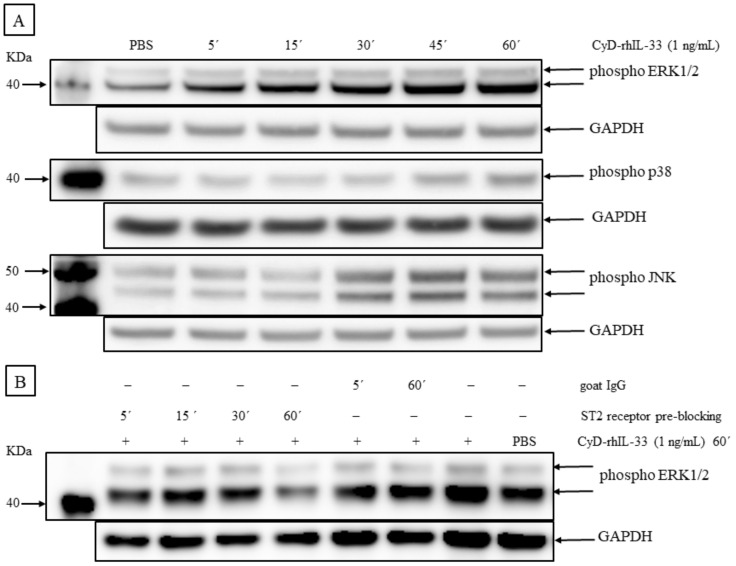
IL-33 upregulates MAP kinase activity. (**A**) Phosphorylation levels of ERK1/2, p38, and JNK were determined by western blot analysis using phospho-specific antibodies to detect Thr202/Tyr204 (ERK1/2), Thr183/Tyr185 (JNK), and Thr180/Tyr182 (p38) phosphorylation, when compared to GAPDH. MCC-13 cells were serum-starved for 24 h, and thereafter stimulated with either vehicle (PBS) or 1 ng/mL of CyD-rhIL-33 for different time points (5′, 15′, 30′, 45′, and 60′). (**B**) The blockade of the ST2 receptor interferes with IL-33-induced activation of ERK1/2 activity. Phospho ERK1/2 levels were determined by western blot. MCC-13 cells were serum starved for 24 h. The cells were pre-blocked with goat anti-ST2 antibody (2 ng/mL) for a varying time duration of 5–60 min or with polyclonal normal goat IgG for 5 or 60 min. The cells were then stimulated with CyD-rhIL-33 (1 ng/mL) in the presence of, or without a ST2 receptor antibody, for 60 min. PBS was used as a vehicle control.

IL-33 activates multiple signaling pathways in different cellular systems, including NF-κB and the MAPK signaling cascades ERK, JNK, and p38 [23,41,42,43,44,45]. The effect on these pathways in V− MCC-13 cells was investigated after stimulation with CyD-rhIL-33 (1 ng/mL) for different time periods (5–60 min). The phosphorylation of ERK1/2, JNK, and p38 was assessed by western blotting using different phospho-specific antibodies (Figure 9A). An increase in ERK1/2, p38, and JNK phosphorylation was observed, although with different kinetics and stoichiometry. Robust ERK1/2 phosphorylation was already seen 5 min after stimulation, while p38 and JNK phosphorylation was noticed after 45 min and 30 min, respectively. Pre-blocking the IL-33 receptor ST2L with a specific antibody abrogated the CyD-rhIL-33-induced phosphorylation of ERK1/2 (Figure 9B).

**Figure 10:**
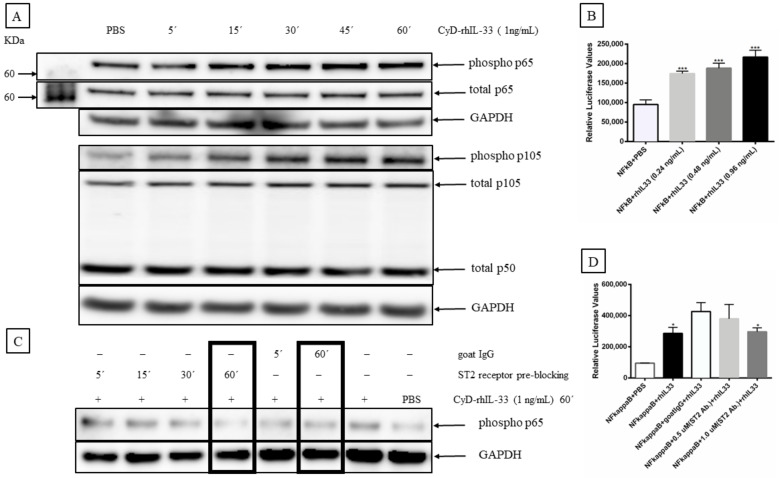
IL-33 upregulates NF-κB activity. (**A**) NF-κB activation was determined by monitoring phosphorylation of the subunit p65 and the p50 precursor p105. MCC-13 cells were serum starved for 24 h, and then stimulated with 1 ng/mL of CyD-rhIL-33 for varying times from 5–60 min. PBS was used as a vehicle control. Relative phospho-p65 and phospho-p105 levels were determined by western blot using phospho-specific antibodies. (**B**) The effect of rhIL-33 on NF-κB-mediated promoter activity. NF-κB activity was measured by using a luciferase reporter plasmid containing a minimal promoter (TATA box) and a NF-κB-responsive element. Cells were stimulated with 0.24, 0.48, and 0.96 ng/mL of rhIL-33 for 4 h. Luciferase values were normalized to total protein. (**C**) The effect of ST2 receptor blocking on the IL-33-induced activation of NF-κB. Phospho-p65 levels were determined by western blotting. MCC-13 was serum starved for 24 h, and then pre-blocked with goat anti-ST2 antibody (2 ng/mL) or normal goat IgG for a varying time duration (5–60 min). The cells were then stimulated with CyD-rhIL-33 (1 ng/mL) for 60 min. PBS was used as a control. (**D**) Anti-ST2 antibody ablates the IL-33-induced activation of NF-κB. Cells were transfected with the luciferase reporter plasmid with an NF-κB responsive promoter and to CyD-rhIL-33 (1 ng/mL) in the presence of, or without, anti-ST2 antibody (0.5 or 1 μg/mL) for 4 h. Goat IgG was used as a control. Each bar represents the average of three independent parallels. Luciferase values were corrected for protein concentration of the samples. * *p* ≤ 0.05, *** *p* ≤ 0.001.

NF-κB is an inducible transcription factor that responds to different inflammatory mediators, and whose activity is regulated through several mechanisms, both in the nucleus and cytoplasm [46]. The most prevalent form of NF-κB is the p65:p50 heterodimer [47]. To evaluate the effect of IL-33 on NF-κB activation, we stimulated MCC-13 cells with 1 ng/mL of CyD-rhIL-33 protein and monitored the phosphorylation status of p65 and the p50 precursor p105 at different time points (5–60 min). An increase in p65 and p105 phosphorylation was observed following stimulation with CyD-rh-IL33 (Figure 10A). Furthermore, pre-incubation with a ST2L receptor-specific antibody inhibited the IL-33-induced phosphorylation of p65 (Figure 10C). To support our western blot findings, we performed a NF-κB promoter reporter assay. The MCC-13 cells were co-transfected with the CyD-IL-33 expression plasmid and a luciferase reporter plasmid with an NF-κB responsive promoter. We determined that CyD-rhIL-33 activated p65 promoter activity with increasing CyD-IL-33 plasmid concentration (Figure 10B), and the promoter activity was inhibited by blocking ST2L receptor using an anti-ST2 antibody (Figure 10D).

### 2.8. IL-33 and Its Receptors, ST2/IL1RL1, and IL1RAcP Are Expressed in MCC Tissue Samples

**Figure 11:**
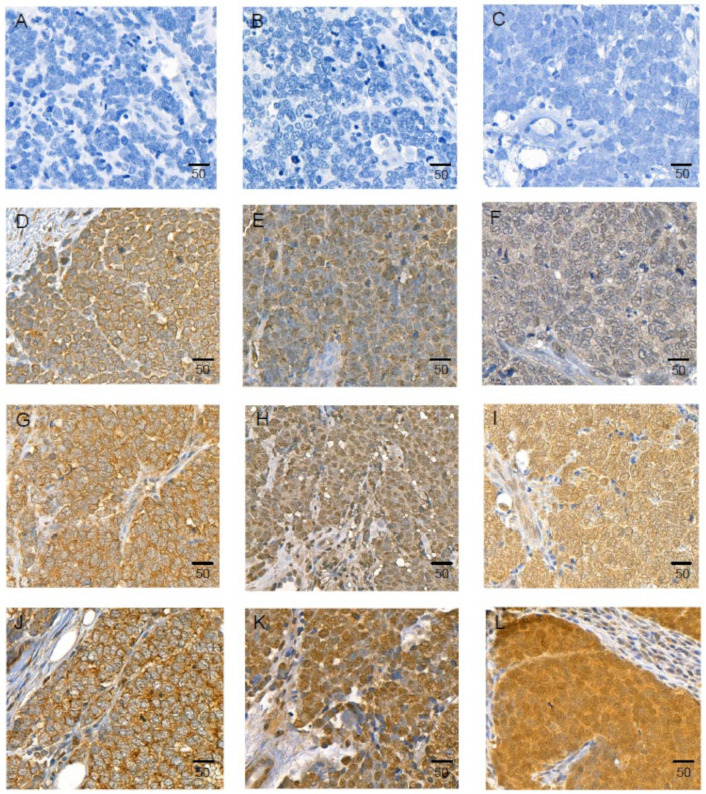
Immunohistochemical staining of IL-33, ST2/IL1RL1, and IL1RAcP in MCC. Isotype control and low, intermediate, and high expression of IL-33 (**A**,**D**,**G**,**J**), ST2/IL1RL1 (**B**,**E**,**H**,**K**), IL1RAcP (**C**,**F**,**I**,**L**), respectively. Scale bar = 50 μm.

The difference in the expression levels of IL-33 in V− and V+ MCC cell lines (Figure 2) caused us to examine IL-33 levels in MCC tumors. A total of 23 primary cutaneous MCCs were immunohistochemically stained for LT, CK20, IL-33, ST2/IL1RL1, and IL1RAcP (Figure 11A–J). Fifteen of twenty-three (65.2%) demonstrated an intranuclear positivity for LT. All of the 23 MCC tissue samples displayed a uniform positivity for CK20 (dot-like cytoplasmic), IL-33 (cytoplasmic), ST2/IL1RL1 (nuclear), and IL1RAcP (nuclear and cytoplasmic).

Associations of IL-33, ST2/IL1RL1, IL1RAcP expression, clinicopathological factors (size, sex, age, stage [1 and 2 vs. 3 and 4], LT expression, presence of MCPyV DNA), and survival was investigated further with tissue microarray in a series of 138 MCC patient samples (Figure 11). The vast majority of the MCC samples analyzed expressed IL-33 (137/138) and IL1RAcP (134/138). IL-33 expression did not show statistically significant correlation with the investigated clinicopathological factors (all *p*-values > 0.05; Supplementary Table S4). ST2/IL1RL1 expression associated with presence of MCPyV DNA (*p* = 0.01), and IL1RAcP expression associated with LT- and DNA-positivity, and less frequently in tumors located in the head (all *p*-values < 0.04; Supplementary Table S4). Any associations between IL-33, ST2/IL1RL1, and IL1RAcP expression and MCC-specific or overall survival were not found.

### 2.9. IL-33, sST2, and sIL1RAcP Measurement in Human Patient Plasma Samples

**Figure 12:**
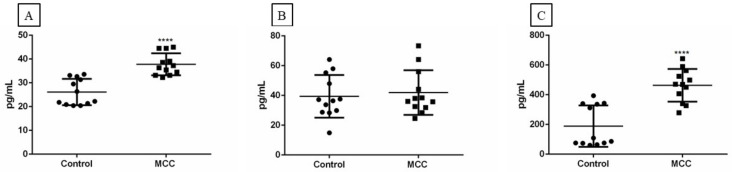
Detection of IL-33 (**A**), sST2/IL1RL1 (**B**), and sIL1RAcP (**C**) levels in plasma samples from MCC patients (*n* = 12) and healthy controls (*n* = 12) by ELISA. **** *p* ≤ 0.0001.

The expression of IL-33, ST2, and IL1RAcP in MCC tissue samples, together with our in vitro data, suggest that the IL-33/ST2-IL1RAcP axis may play a role in MCC. This prompted us to compare IL-33, sST2/IL1RL1, and sIL1RAcP levels in the plasma of 12 healthy subjects and 12 MCC patients. The minimum level of 3.1 pg/mL, 31.3 pg/mL, and 31.2 pg/mL was set as detectable for IL-33, sST2, and sIL1RAcP, respectively (recommended by the supplier). The mean IL-33 plasma values were 26.14 ± 5.53 pg/mL and 37.81 ± 4.64 pg/mL for control and MCC patients, respectively (Figure 12A). The mean sST2/IL1RL1 plasma values were 39.36 ± 14.32 pg/mL and 41.92 ± 14.96 pg/mL for control and MCC patients, respectively (Figure 12B). Similarly, the mean sIL1RAcP plasma values were 189.20 ± 139.288 pg/mL and 463.45 ± 110.28 pg/mL for healthy controls and MCC patients, respectively (Figure 12C). Significantly higher IL-33 and IL1RAcP levels were found in the MCC patients compared with the healthy individuals.

## 3. Discussion

In the present study, we investigated the differential expression of 84 inflammatory mediators in V+ and V− MCC cell lines and explored a possible role for MCPyV T-ags in the regulation of these inflammatory mediators. Previous studies reported that MCPyV sT downregulates IL-2, IL-8, CCL20, and CXCL9 expression in MCC-13 cells [48], while MCPyV LT, tLT (LT339), and sT upregulate IL-1β, IL-6, IL-8, CXCL1, and CXCL6 in hTERT-immortalized BJ human foreskin fibroblasts [49]. A previous study by our group also found that MCPyV LT and tLT upregulate CCL17/TARC expression in a V+ MCC (MKL-2) cell line, as compared to a V− MCC (MCC-13) cell line [27]. A recent study reported that MCPyV infection of human dermal fibroblasts triggers the expression of the inflammatory cytokines IL-1β, IL-6, IL-8, and TNFα [50].

In this work, we focused on IL-33, one of the cytokines with higher transcript levels in the V+ MCC cell lines compared to V− MCC cell lines. Previous studies demonstrated an enhanced expression of IL-33 after viral infections (including tumor viruses), and in immunoresponses in different malignancies [20,28,29,30,31,32,33]. Western blot analysis confirmed higher IL-33 levels in V+ MCC cells than in V− MCC cells and HDF, although IL-33 expression levels did not vary between V− and V+ MCC patient samples. However, increased expression of IL-33 receptors ST2/IL1RL1 and IL1RAcP in the tumor cells was associated with the presence of MCPyV. Comparing the levels of IL-33 and its receptors in the plasma of MCC patients and healthy individuals confirmed a higher IL-33 and IL1RAcP in the patients, whereas no significant difference in ST2 levels was found between the two cohorts. Previous studies have shown elevated IL-33 plasma levels in lung cancer [51], breast cancer [52,53], gastric cancer [54], and advanced pancreatic ductal adenocarcinoma [55].

To understand the enhanced level of IL-33 in V+ MCC cells compared with V− MCC cells, we investigated a possible involvement of MCPyV LT and sT in IL-33 expression regulation. We found that both viral proteins stimulated the promoter activity of the IL-33 receptors, ST2/IL1RL1 and IL1RAcP, and IL-33 protein expression was increased in MCC-13 cells transiently transfected with MCPyV T-ags. The regulation of IL-33 expression in MCC is not understood. Deletion of the DNA-binding domain of LT or mutations in the LT binding GRGGC motifs did not abrogate LT-induced activation of the IL-33 promoter, thereby suggesting that no direct binding of LT to DNA is required to activate the IL-33 promoter. Additionally, nuclear localization may not be required, as the tLT variant LT_MKL-2_, which lacks a nuclear localization domain [56], was still able to stimulate the *IL-33* promoter. However, we cannot exclude that LT_MKL-2_, in a complex with other proteins, is transported into the nucleus. Experiments with truncated *IL-33* promoters revealed that the sequences proximal to the transcription start site can still mediate T-ags-induced activation of the *IL-33* promoter. This suggests that either T-ags interact with components of the initiation complex or mediate its effect to transcription factors that bind in this region. Indeed, the LT of polyomaviruses can interact with general transcription factors such as TFIIB, TBP, TAFIIs, and the B subunit of RNA polymerase II, and with the transcription factors DP1, SP1, p53, and p300 [34,57]. The *IL-33* −160/+50 promoter fragment contains putative binding motifs for these transcription factors using the PROMO TRANSFAC program [58,59]. Gorbacheva et al. [60] showed that an increase in the activity of SP1 was associated with elevated levels of *IL-33* in breast and lung cancers [60]. MCPyV sT also potentiated *IL-33* promoter activity. Polyomavirus sT can interact with DP1 and can regulate the activity of transcription factors such as Elk-1, Ets-1, and AP1 through activation of the MAPK pathway [57,61]. Further studies are required to unveil the exact mechanism by which MCPyV LT and sT increase *IL-33* promoter activity.

IL-33 possesses transcriptional regulatory functions associated with a homeodomain-like, helix-turn-helix motif [62,63,64]. Nonetheless, the activity of IL-33 is regulated by proteolytic cleavage. Inflammatory proteases from neutrophils (proteinase 3, elastase, and cathepsin G) [65] and mast cells within the tumor microenvironment (chymase, tryptase, and granzyme B) [21] can process full-length IL-33 into shorter mature forms (18–21 kDa), with a 10- to 30-fold higher amount of activity [66,67]. Based on the diverse activity of these proteolytic variants, we studied a possible autocrine effect of IL-33 and IL-33 fragments on MCPyV early and late promoter activity. The cytokine domain of IL-33 (CyD-IL-33) upregulated, whereas full-length IL-33 downregulated the activity of both promoters. The inhibitory effect of FL-IL-33 was mediated by its nuclear domain. Since both IL-33 receptors occur in a soluble form, we also examined their effect on MCPyV promoter activity. sST2 increased, whereas sIL1RAcP suppressed both early and late promoter activity. This suggests that during disease, sST2 may upregulate MCPyV LT and sT expression, and contribute to MCPyV−induced development of MCC. On the other hand, sIL1RAcP may repress LT and sT expression.

Earlier reports have demonstrated that IL-33 activates the MAPK pathways in various cancer cell lines [23,41,68,69,70], and showed an IL-33/ST2-dependent NF-κB activation [45,71,72]. We confirmed that IL-33/ST2 activated both pathways in MCC cells; this may explain the stimulatory effect on the MCPyV promoters, which contains putative binding sites for NF-κB and for ERK1/2 MAPK-regulated transcription factors, such as AP1, ATF/CREB, c-MYC, ELK1, and SP1 [73,74].

**Figure 13:**
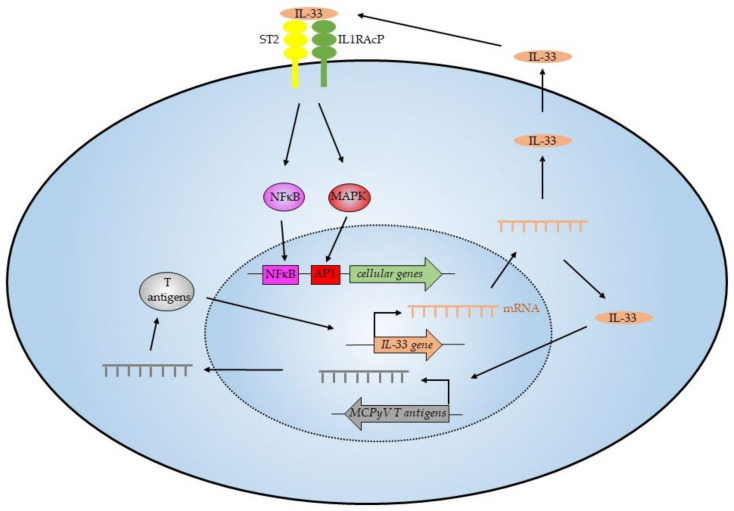
The IL-33/ST2 axis in MCPyV−positive MCC. The T-antigens of MCPyV stimulate expression of IL-33. Excreted IL-33 can bind to its receptors ST2 and IL1RAcP on the cell surface and this will activate the NF-κB and MAPK pathways, subsequently resulting in the expression of NF-κB and MAPK-responsive genes. IL-33 also increases the expression of the MCPyV T-antigens, and this will kindle an autostimulatory loop of IL-33 expression.

In conclusion, our results demonstrate that MCPyV LT and sT are linked to an altered expression of IL-33 and its receptors, ST2/IL1RL1 and IL1RAcP. The expression of IL-33 and its receptor ST2/IL1RL1 may constitute an autocrine or paracrine survival loop, which contributes to the growth and survival of MCC (Figure 13). Several studies underscore a versatile role of the IL-33/T2-IL1RAcP pathway in the tumor microenvironment and tumorigenesis [20,29], thus making these proteins attractive therapeutic targets and potential biomarkers in MCC. Proof-of-concept is provided in clinical studies with the IL-33 antibody etokimab, and with the ST2 antibody astegolimab showing improvement in patients with atopic dermatitis [75], a peanut allergy [76], or severe asthma [77]. Therapeutic strategies for targeting IL-33/ST2 signaling for the treatment of various inflammatory diseases are evolving [78,79]. However, additional studies are required to establish that blocking the IL33/ST2 axis may have therapeutic potential for MCC treatment.

## 4. Materials and Methods

### 4.1. Materials

The primary antibodies used in this study are displayed in Supplementary Table S1. The recombinant proteins include recombinant human full-length IL-33 (FL-rhIL-33) (cat.#TP760633, Origen Technologies, Inc. Rockville, MD, USA) and recombinant human cytokine-domain IL-33 (CyD-rhIL-33) (cat.# 3625-IL, R&D systems, Abingdon, UK).

### 4.2. Plasmids

The empty expression plasmid pcDNA3.1(+) was purchased from Invitrogen. Full-length LT expression plasmid pcDNA6.MCV.cLT206.V5_CM2B4 was purchased from Addgene (Cambridge, MA, USA), while the expression plasmid for LT_MKL-1_, LT_MKL-2_, and LT_MS-1_ were constructed by site-directed mutagenesis using the original pcDNA6.MCV.cLT206.V5_CM2B4 plasmid and have been previously described [27]. pNFκB-LUC plasmid was obtained from Clontech (Takara Bio, Mountain View, CA, USA), and MCPyV sT-FLAG was a kind gift from Andrew Macdonald [80]. IL-33, ST2 (IL1RL1), and IL1RAcP expression plasmids were generated by cloning amplified cDNA into pCMV3_His(N)_FLAG(C). The plasmids pGL3_IL33(−1050/+50), pGL3_ST2L(−100/+82), pGL3_sST2(−499/+100), pGL3_IL1RAP(−1397/+182), and pGL3_IL1RAP(−517/+182) were amplified by PCR of the promoter sequence from genomic DNA purified from blood, and cloned into the luciferase reporter plasmid pGL3-basic (Promega). Luciferase reporter plasmids containing truncated versions of the *IL-33* promoter were generated by site-directed mutagenesis using complementary primers containing a *KpnI* restriction enzyme motif, and additional *KpnI* site was introduced in the pGL_IL33(−1050/+50) vector. The plasmid was then digested with *KpnI* and re-ligated. Site-directed mutagenesis was also applied to destroy each of the two putative LT motifs in the *IL-33* promoter. A double *IL-33* promoter mutant, in which both LT binding motifs were destroyed, was also generated. The primers used for cloning are shown in Supplementary Tables S2 and S3. All constructs were verified by sequencing.

### 4.3. Cell Lines and Human Tissue Samples

MCC-13, MCC-26, UISO, MKL-1, MKL-2, and MS-1 cell lines were kindly provided by Dr. Baki Akgül (University of Cologne, Cologne, Germany). The WaGa cell line was a kind gift from Dr. JC Becker (University Duisburg-Essen, Duisburg, Germany). MCC-13, MCC-26 [81], and UISO [82] are V− MCC cell lines, whereas MKL-1 [83], MKL-2, WaGa [84,85], and MS-1 [86] are V+ MCC cell lines. The immortalized human dermal fibroblast cell line fHDF/TERT166 was purchased from Evercyte GmbH (Vienna, Austria) and kept in FibroUp (Evercyte; Wien, Austria, cat. no. MHT-008), 1:1 DMEM/Ham’s F-12 (Biochrom, Nuremberg, Germany; cat. no. F4815), 2 mM Glutamax^TM^-I (ThermoFisher Scientific, Waltham, MA, USA; cat. no. 35050061), 10% FBS, and 100 mg/mL G418 (Invivogen, San Diego, CA, USA; cat. no. ant-gn5). Both V+ and V− cells were grown in RPMI-1640 containing 10% FBS, streptomycin (100 μg/mL), and penicillin (100 U/mL). The cells were kept in a humidified CO_2_ incubator at 37 °C. Human MCC tissues were obtained during 2000–2015 from the St. Olavs University Hospital Trondheim, Norway, in accordance with the ethical approval from the Regional Ethical Committee (REK NORD application number 2016/988). Plasma samples from 12 MCC patients were obtained with consent, in accordance with the ethical approval from the Ethics Committee of Karolinska Institutet, Sweden (2010/1092-31/3). Plasma samples from healthy individuals were obtained with consent and approved by the Regional Committee for Medical and Health Research Ethics, Northern Norway (REK151884).

Tumor tissues and clinical data was available from 168 patients diagnosed with MCC in Finland between 1 January 1979, and 31 December 2013 (Supplementary Table S4). Patients were identified and clinical data were obtained from hospital case records, and the Finnish Cancer Registry and FFPE tissue samples were collected from the pathology archives. Tissue microarrays were constructed and expression of MCPyV LT and the presence of MCPyV DNA in tumor tissue was detected as described in detail earlier [87]. The study protocol was approved by the Ethics Committee of Helsinki University Central Hospital (HUS/1455/2017). The Ministry of Health and Social Affairs granted permission to collect patient data (STM/398/2005) and the National Authority for Medicolegal Affairs to collect tissue samples (4942/05.01.00.06/2009).

### 4.4. Transfection

Cells were seeded out in 6- and 12-well cell culture plates with a total number of 2 × 10^5^ and 1.0 × 10^5^, respectively. At the time of transfections, the cells were approximately 80–90% confluent, and jetPRIME (Polyplus-transfection SA, Illkirch, France) was used to transfect all plasmids, according to the manufacturer’s instructions. For gene promoter analysis, the cells were transfected with polyethylenimine (PEI linear MW25000; transfection grade, cat.# 3966–1, Polysciences, Warrington, PA, USA). DNA was mixed with 150 mM NaCl, and a mixture of PEI:150 mM NaCl was then added to the DNA at a ratio DNA:PEI of 1:4. This mixture was incubated for 15 min at room temperature, and then carefully added to the cells. The medium containing the transfection mixture was replaced 4 h later. A total of 2 μg DNA per well in a 6-well plate, and 1 μg of DNA per well in a 12-well plate was used to transfect cells. All experiments were performed 24 h after transfection.

### 4.5. RNA Extraction

RNA extraction was performed using the RNeasy^®^ Plus Mini kit (Qiagen, Hilden, Germany), according to the manufacturer’s protocol. For RNA yield and quality, A260/A280 and A260/A230 ratios were analyzed with the Nano-Drop^®^ ND-2000 spectrophotometer (NanoDrop Technologies, ThermoFisher Scientific, Waltham, MA, USA).

### 4.6. cDNA Construction and Quality Control

The iScript™ cDNA Synthesis Kit (BioRad, Hercules, CA, USA) was used to generate cDNA. A total of 1–2 μg of RNA was converted to cDNA, according to the manufacturer’s instructions. To determine the presence of genomic DNA contamination, PCR with the housekeeping APRT primers (5′-CCCGAGGCTTCCTCTTTGGC-3′ and 5′-CTCCCTGCCCTTAAGCGAGG-3′) was performed. In case of genomic DNA contamination in the purified RNA sample, RT-PCR will generate an 800 bp fragment, whereas RT-PCR of RNA gives a 300 bp amplicon [88]. PCR products were visualized on a 1% agarose gel stained with GelRed™ Nucleic Acid Gel Stain (Biotium, Cambridge Bioscience, Bar Hill, UK).

### 4.7. RT^2^ Profile PCR Array

Human cytokine and receptor gene transcription was measured using the human RT^2^ Profiler PCR Inflammatory Cytokines and Receptors Array (PAHS-011ZA, SABiosciences, Qiagen). The cDNA was added into RT^2^ SYBR Green Mastermix and amplified using the Light Cycler 96 (Roche Diagnostics, Indianapolis, IN, USA) with 95 °C for 10 min, followed by an alteration of 95 °C (15 s) and 60 °C (1 min) for 45 cycles. All data were collected by the Light Cycler 96 SW 1.1 software and analyzed using the Gene Glob PCR Array Data Analysis Web Portal (Qiagen, Hilden, Germany). For considering a gene differentially expressed, we used a 2-fold change as a cut-off.

### 4.8. Luciferase Assays

For luciferase assays, approximately 24 h after transfection, cells were lysed in 100 μL Luciferase Assay Tropix Lysis solution (ThermoFisher Scientific) with freshly added 0.5 mM DTT. Cells were scraped, transferred to Eppendorf tubes, and then centrifuged for 3 min at 12,000× *g*. Twenty μL of the supernatant was added to a 96-well microtiter plate containing 50 μL luciferase buffer (Promega, Madison, WI, USA). The CLARIOstar Plus Microplate reader (BMG Labtech, Ortenberg, Germany) was used to measure relative luciferase units (RLU). Each experiment was repeated three times with three parallel samples for each experiment. Luciferase values were corrected for the total protein concentration, which was measured using the MN protein quantification assay (Macherey-Nagel GmbH, Düren, Germany).

### 4.9. Immunoblotting

Western blot was performed by separating protein samples on 4–12% NuPAGE Bis-Tris Mini Gels (Invitrogen Life Technologies, Carlsbad, CA, USA), according to the manufacturer’s protocol. Proteins were blotted onto a 0.45 μm PVDF membrane (Millipore, Billerica, MA, USA), and blocking was performed using TBS-T (TBS with 0.1% Tween-20; Sigma Aldrich) containing 5% (*w*/*v*) dried skimmed milk for 1 h. The protein was probed by incubating the membrane with the primary antibody overnight at 4 °C. After washing the membrane 3 times with TBS-T, an appropriate secondary antibody was added for 1 h at room temperature. After 2 washes with TBS-T and 2 washes with washing buffer, antigen-antibody complex was visualized using SuperSignal™ West Pico Chemiluminescent Substrate (Cat.#34080 Thermo Fisher Scientific, Rockford, IL, USA). The Magic-Mark™ Western standard from Invitrogen Life Technologies was used to estimate the molecular mass of the detected proteins. Densitometric analysis of western blots was performed by using imageJ (NIH, Bethesda, MD, USA).

### 4.10. Immunohistochemistry

Formalin-fixed and paraffin-embedded tissue sections were deparaffinized in xylene and graded alcohols, and then hydrated and washed in PBS. After antigen retrieval in a sodium citrate buffer (pH 6) in a microwave oven, the endogenous peroxidase was blocked by 0.3% H_2_O_2_ for 15 min. Sections were incubated overnight at 4 °C with the primary antibodies against MCPyV−LT, IL-33, ST2/IL1RL1, IL1RAcP, and CK20 (Supplementary Table S1). As a secondary antibody, either the anti-rabbit-HRP Signalstain (R) DAB Substrate kit (Cat.# 8059S Cell Signaling Technologies, Beverly, MA, USA) or anti-mouse EnVision-HRP (Dako, Agilent Technologies, Inc., Santa Clara, CA, USA) was used. A matched isotype control was used as a control for nonspecific background staining. Staining intensity was scored as negative, low, intermediate, or strong when >20% of tumor cells showed protein expression.

### 4.11. Plasma Levels of IL33, ST2/IL1RL1, and IL1RAcP in MCC Patients and Healthy Controls

Plasma concentrations of IL-33, ST2IL1RL1, and IL1RAcP were measured by enzyme-linked immunosorbent assay (ELISA) using Human ST2/IL-33R DuoSet ELISA, Human IL-1 RAcP/IL-1 R3 DuoSet ELISA and Human IL-33 Quantikine ELISA Kit (R&D systems, Abingdon, U.K), according to the manufacturer’s protocol. The assay ranges for IL-33, ST2/IL1RL1, and IL1RAcP were 3.1–200 pg/mL, 31.2–2000 pg/mL, and 31.2–2000 pg/mL, respectively.

### 4.12. Statistical Analysis

The RT^2^ Profiler PCR Array data analysis version 3.5 (http://dataanalysis.sabiosciences.com/pcr/arrayanalysis.php accessed on 21 January 2020) was used for inflammatory cytokines and receptor data analysis. For the analysis, values with a fold change ≥2 and a *p*-value less than 0.05 were considered significant. GraphPad Prism 6 software was used for statistical analysis and graph design. The Student’s *t*-test was used to compare differences between the experimental and control group, with a *p*-value < 0.05 considered statistically significant.

## Supplementary Materials

The following are available online at https://www.mdpi.com/article/10.3390/ijms23073702/s1.
