# Supplementary material for: The Merkel Cell Polyomavirus T-Antigens and IL-33/ST2-IL1RAcP Axis: Possible Role in Merkel Cell Carcinoma
Source: Int J Mol Sci. 2022 Mar 28;23(7):3702. doi: 10.3390/ijms23073702 (PMC8998536; doi:10.3390/ijms23073702)
Supplement: Supplementary file 1 [file ijms-23-03702-s001.zip › ijms-1605576-supplementary.pdf]

Supplementary Table S1. Primary antibodies used in this study

Antibody	Supplier	Method*
monoclonal mouse MCPyV LTA	Sc-136172, Santa Cruz Biotechnologies	IHC
mouse IL33 monoclonal antibody (Nessy-1)	cat.#ALX-804-840, Enzo	IHC
Rabbit CK-20 monoclonal antibody (SP33)	cat.#790-4431, Roche	IHC
mouse IL33 monoclonal antibody (4E9)	cat.#ab72844, abcam	WB
rabbit anti-ST2	cat.# PRS3363, Sigma-Aldrich	IHC
rabbit IL1RAcP/IL-1R3 polyclonal antibody	cat.#NBP2-16946, novusbio	WB
rabbit polyclonal IL-1 RAcP/IL-1 R3 antibody	cat.# NBP1-76464	IHC
monoclonal rabbit keratin 20	cat.# 13063, Cell Signaling, Danvers, MA, USA	
monoclonal rabbit Phospho-p44/42 MAPK (Erk1/2) (Thr202/Tyr204)	cat.#4370, Cell Signaling	WB
monoclonal rabbit p44/42 MAPK (Erk1/2)	cat.#4695S, Cell Signaling	WB
Phospho-SAPK/JNK (Thr183/Tyr185) (81E11) rabbit mAb	cat.#4668, Cell Signaling	WB
SAPK/JNK rabbit polyclonal antibody	cat.#9252, Cell Signaling	WB
Phospho-p38 MAPK (Thr180/Tyr182)	cat.#9211, Cell Signaling	WB
Phospho-NF-κB p65 (Ser536) (93H1) Rabbit mAb	cat.##3033, Cell Signaling	WB
Phospho-NF-κB p105 (Ser933) (18E6)	cat.#4806S, Cell Signaling	WB
rabbit anti-GAPDH	cat.# G9545, Sigma Aldrich, St. Louis, MO, USA	WB
goat ST2/IL-33 R polyclonal antibody	cat.#AF523, R&D systems	B
polyclonal normal goat IgG control	cat.#AB-108-C, R&D systems	B.Ctrl

*IHC: immunohistochemistry; WB: western blot; B: blocking; B.Ctrl: blocking control

Supplementary Table S2. List of primers used to clone expression plasmids in pCMV3_His(N)_FLAG(C)*.

Sr.#	Plasmid Name	Forward Primer(5'>3')	Reverse Primer (5'>3')
1	IL33(1-270aa)	GGTAGCGCTAGCATGAAGCCTAAAATGAAG	TCCATaACCGGTAGAGTTTCAGAGAGCTTA
2	IL33(112-270aa)	GGTAGCGCTAGCATGAGTATCACAGGAATT	TCCATaACCGGTAGAGTTTCAGAGAGCTTA
3	IL33(1-65aa)	GGTAGCGCTAGCATGAAGCCTAAAATGAAG	CCCTAAACCGGTAGGGTGGTTTCTCTCCTA
4	IL33(1-111aa)	GGTAGCGCTAGCATGAAGCCTAAAATGAAG	CCCTAAACCGGTAGTGAATCATGAAGTGCT
5	IL33(1-65,112-270aa)	Synthesize from GeneScript	
6	IL33(66-111aa)	GGTAGCGCTAGCATGAAAAGGCCTTCACTG	CCCTAAACCGGTAGTGAATCATGAAGTGCT
7	IL33(66-270aa)	GGTAGCGCTAGCATGAAAAGGCCTTCACTG	TCCATaACCGGTAGAGTTTCAGAGAGCTTA
8	ST2L*	GGCTAGCATGGGGTTTTGGATCTTAG	CACCGGTAGTTGCTTCTGGGCAGCC
9	sST2*	GGCTAGCATGGGGTTTTGGATCTTAG	CACCGGTAGGAAACACTCCTTACTTGG
10	IL1RAcP.L *	GGCTAGCATGACACTTCTGTGGTGTG	CACCGGTAGTACATTTTTCAAAGATGAATAC
11	sIL1RAcP*	GGCTAGCATGACACTTCTGTGGTGTG	CACCGGTAGCTGACCGCATCTATTACC

* His(N): His Tag on N-terminal; FLAG(C): FLAG tag on C-terminal; L: membrane bound receptor; s: soluble form of receptor

Supplementary Table S3. List of primers used to synthesize luciferase promoter plasmids.

Sr.#		Forward Primer(5'>3')	Reverse Primer(5'>3')
1	pGL3_IL33(-1050/+50)	AGTCACGGTACCATTAAAGGGGATTACTGCACAGAAAATGAAACAAC	AGTCACAAGCTTTTTCTTCCCTGAAGAGCTGCAGCTCTGTGTTCGGAGG
2	pGL3_IL33 (-656+50)	CCAAGTCTTAAAAGAGCGGTACCTCATCCC	GGGATGAGGTACCGCTCTTTTAAGACTTGG
3	pGL3_IL33 (-598+50)	GAGGGTGAGTGGTACCAAAATTTCTCATGAGG	CCTCATGAGAAATTTTGGTACCACTCACCCTC
4	pGL3_IL33 (-460/+50	GGGGAGCAGGTACCCCCGTCAGATATGTTGG	CCAACATATCTGACGGGGGTACCTGCTCCCC
5	pGL3_IL33 (-210/+50	GTTCAACTCCAAAGGGTACCAAAACATAAAGTTTAGG	CCTAAACTTTATGTTTTGGTACCCTTTGGAGTTGAAC
6	pGL3_IL33 (-160/+50	GCTGGTTTAAGGTACCCAGATGGAGGGAGG	CCTCCCTCCATCTGGGTACCTTAAACCAGC
7	mutLT1	CTTTTTTCTGCTCCGCGTTTGTTTCAAG	CCTTGAAACAAACGCGGAGCAGAAAAAAG
8	mutLT2	GAATGGATGTAGATCTTTGCTAGGGTAAC	GTTACCCTAGCAAAGATCTACATCCATTC
9	mutLT3	GCCAAAACATAAAGTTTAGCGCCAGAAGAAGAATC	GATTCTTCTTCTGGCGCTAAACTTTATGTTTTGGC
10	pGL3_ST2L(-100/+84)	GGCTCGAGGGTGAAAACTGCAGTTAACATTTG	CCAAGCTTCTGCCCACAGTTTCACAACTCAGAAAGCCA
11	pGL3_sST2(-499/+100)	TTTGGTACCTGGGATGGGGTGGAGTCACATTC	TTTCTCGAGGATAAGTAATTGATGCTTACC
12	pGL3_IL1RAcP(-1397/+182)	GGTACCTGACAATGTTGTGAGCAGGTG	CCAAGCTTTTCTGCGGCTTGGTGACTTCT
13	pGL3_IL1RAcP(-517/+182)	GGTACCGGTGGTTGCGAAAATCGTGCA	CCAAGCTTTTCTGCGGCTTGGTGACTTCT

Supplementary Table S4. Association of IL-33, ST2/IL1RL1 and IL1AcP expression with clinicopathological factors in Merkel cell carcinoma.

Variable	IL33				ST2/IL1RL1				IL1AcP			
	**0+1**	**2**	**3**	***P***	**0+1**	**2**	**3**	***P***	**0+1**	**2**	**3**	***P***
	n=20 (%)	n=47 (%)	n=51 (%)		n=43 (%)	n=44 (%)	n=39 (%)		n=44 (%)	n=44 (%)	n=29 (%)	

MCPyV DNA				.165				.010				.011
negative	4 (12.1)	19 (57.6)	10 (30.3)		16 (88.5)	10 (30.3)	7 (21.2)		20 (64.5)	7 (22.6)	4 (12.9)	
positive	5 (8.8)	23 (40.4)	29 (50.9)		13 (19.7)	26 (39.4)	27 (40.9)		20 (31.7)	30 (47.6)	13 (20.6)	
NA	11	5	12		14	8	5		4	7	12	
MCPyV LT				.622				.073				.040
negative	5 (11.4)	19 (43.2)	20 (45.5)		20 (44.4)	15 (33.3)	10 (22.2)		21 (50)	10 (23.8)	11 (26.2)	
positive	13 (18.1)	28 (38.9)	31 (43.1)		20 (25.6)	29 (37.2)	29 (37.2)		22 (29.7)	34 (45.9)	18 (24.3)	
NA	2	0	0		3	0	0		1	0	0	
Sex				.576				.319				.163
Female	14 (17.1)	35 (42.7)	33 (40.2)		27 (31.0)	34 (39.1)	26 (29.9)		33 (40.7)	32 (39.5)	16 (19.8)	
Male	6 (16.7)	12 (33.3)	18 (50.0)		16 (41.0)	10 (25.6)	13 (33.3)		11 (30.6)	12 (33.3)	13 (36.1)	
Location				.638*				.464*				.003*
Head	11 (17.7)	24 (38.7)	27 (43.5)		25 (37.9)	24 (36.4)	17 (25.8)		33 (52.4)	19 (30.2)	11 (17.5)	
Limb	6 (14.6)	19 (46.3)	16 (39.0)		13 (30.2)	15 (34.9)	15 (34.9)		6 (15.0)	21 (52.5)	13 (32.5)	
Trunk	0 (0)	4 (40.0)	6 (60.0)		2 (16.7)	4 (33.3)	6 (50.0)		4 (36.4)	4 (36.4)	3 (27.3)	
NA	3	0	2		3	1	1		1	0	2	
Stage #				.523				.149				.642
1 and 2	9 (12.0)	31 (41.3)	35 (46.7)		21 (27.6)	32 (42.1)	23 (30.3)		26 (35.1)	31 (41.9)	17 (23.0)	
3 and 4	4 (20.0)	6 (30.0)	10 (50.0)		10 (50.0)	5 (25.0)	5 (25.0)		5 (27.8)	7 (38.9)	6 (33.3)	
NA	7	10	6		12	7	11		13	6	6	
IL33								.036				.224

negative or low					11 (61.1)	3 (16.7)	4 (22.2)		6 (37.5)	7 (43.8)	3 (18.8)	
intermediate					15 (34.9)	15 (34.9)	13 (30.2)		19 (44.2)	17 (39.5)	7 (16.3)	
high					10 (20.4)	21 (42.9)	18 (36.7)		13 (27.7)	17 (36.2)	17 (36.2)	
NA					7	4	4		6	3	2	
ST2/IL1RL1				.036					19 (61.3)	8 (25.8)	4 (12.9)	.016
negative or low	11 (30.6)	15 (41.7)	10 (27.8)						12 (29.3)	16 (39.0)	13 (31.7)	
intermediate	3 (7.7)	15 (38.5)	21 (53.8						9 (24.3)	18 (48.6)	10 (27.0)	
high	4 (11.4)	13 (37.1)	18 (51.4)						4	2	2	
NA	2	4	2									
IL1AcP				.224				.016				
negative or low	6 (15.8)	19 (50.0)	13 (34.2)		19 (47.5)	12 (30.0)	9 (22.5)					
intermediate	7 (17.1)	17 (41.5)	17 (41.5)		8 (19.0)	16 (38.1)	18 (42.9)					
high	3 (11.1)	7 (30.4)	17 (73.9)		4 (14.8)	13 (48.1)	10 (37.0)					
NA	4	4	4		12	3	2					

*Fisher Freeman Halton test. Frequency tables were analyzed with the Chi-square and Fisher-Freeman-Halton tests and continous variables were investigated with the Kruskal-Wallis test. The Kaplan-Meier method was used to estimate survival utilizing the time from the diagnosis of MCC to death from any cause (overall survival) or from MCC (disease-specific survival) and survival between the groups was calculated using the log-rank test.
